# Genotype–Phenotype Correlations in Duchenne and Becker Muscular Dystrophy Patients from the Canadian Neuromuscular Disease Registry

**DOI:** 10.3390/jpm10040241

**Published:** 2020-11-23

**Authors:** Kenji Rowel Q. Lim, Quynh Nguyen, Toshifumi Yokota

**Affiliations:** 1 Department of Medical Genetics, Faculty of Medicine and Dentistry, University of Alberta, Edmonton, AB T6G2H7, Canada; kenjirow@ualberta.ca (K.R.Q.L.); nguyenth@ualberta.ca (Q.N.); 2 The Friends of Garrett Cumming Research & Muscular Dystrophy Canada, HM Toupin Neurological Science Research Chair, Edmonton, AB T6G2H7, Canada

**Keywords:** Duchenne muscular dystrophy, Becker muscular dystrophy, dystrophinopathy, genotype-phenotype correlations, Canadian Neuromuscular Disease Registry, reading frame rule, dystrophin, multiple logistic regression analysis, exon skipping therapy

## Abstract

Duchenne muscular dystrophy (DMD) is a fatal neuromuscular disorder generally caused by out-of-frame mutations in the *DMD* gene. In contrast, in-frame mutations usually give rise to the milder Becker muscular dystrophy (BMD). However, this reading frame rule does not always hold true. Therefore, an understanding of the relationships between genotype and phenotype is important for informing diagnosis and disease management, as well as the development of genetic therapies. Here, we evaluated genotype–phenotype correlations in DMD and BMD patients enrolled in the Canadian Neuromuscular Disease Registry from 2012 to 2019. Data from 342 DMD and 60 BMD patients with genetic test results were analyzed. The majority of patients had deletions (71%), followed by small mutations (17%) and duplications (10%); 2% had negative results. Two deletion hotspots were identified, exons 3–20 and exons 45–55, harboring 86% of deletions. Exceptions to the reading frame rule were found in 13% of patients with deletions. Surprisingly, C-terminal domain mutations were associated with decreased wheelchair use and increased forced vital capacity. Dp116 and Dp71 mutations were also linked with decreased wheelchair use, while Dp140 mutations significantly predicted cardiomyopathy. Finally, we found that 12.3% and 7% of DMD patients in the registry could be treated with FDA-approved exon 51- and 53-skipping therapies, respectively.

## 1. Introduction

Duchenne muscular dystrophy (DMD) is the most common inherited neuromuscular disorder worldwide, affecting approximately 20 per 100,000 male births (1:5000) [1,2]. DMD is an X-linked recessive disorder that is characterized by progressive body-wide muscle degeneration, with proximal muscle weakness starting at 3–5 years and loss of ambulation during the early teens [3,4]. Cardiac and respiratory symptoms often appear during the third decade of life, which eventually lead to death. DMD is primarily caused by mutations in the *DMD* gene that lead to an absence of dystrophin. Dystrophin is a protein responsible for stabilizing muscle cell membranes during contraction–relaxation cycles; its loss increases the susceptibility of muscles to tear during use [5,6,7]. There is a milder form of the disease called Becker muscular dystrophy (BMD), which is caused by mutations in the same gene. However, mutations in BMD patients generally only reduce the amount or functionality of the dystrophin produced, as opposed to the complete absence of dystrophin seen in DMD [8,9,10].

DMD and BMD are part of a group of disorders called the dystrophinopathies, which are all characterized by mutations in the *DMD* gene. Stark differences between the fatal DMD and mild BMD prompt us to understand how differences in genotype (i.e., mutation) impact phenotype (i.e., clinical outcome). This is especially important since there is no cure for DMD at present. To study these genotype–phenotype correlations, among other purposes, dystrophinopathy patient registries were formed by local, national, and international initiatives to collect information on patient clinical outcomes and *DMD* mutations. Perhaps the most extensive of these would be the TREAT-NMD DMD Global Registry [11] and the Leiden Open Variation Database (LOVD) [12,13], each having data from more than 7000 dystrophinopathy patients across the world. Canada in particular has the Canadian Neuromuscular Disease Registry (CNDR), a national patient registry established in 2011 that also contributes to the TREAT-NMD database [14,15]. As of 1 December 2019, with 4310 registrants, dystrophinopathy patients make up the second-largest disease group in the CNDR at 13.3% [15]. Amyotrophic lateral sclerosis has the most number of registered patients at 36.1%; myotonic dystrophy, limb–girdle muscular dystrophy, and spinal muscular atrophy patients make up 10.5%, 5.9%, and 5.3% of CNDR registrants, respectively.

Here, we aimed to evaluate genotype–phenotype correlations specifically in the Canadian DMD/BMD population, using the information on 402 patients from the CNDR. Similar studies have been conducted previously [11,16,17,18,19,20,21,22]; however, most of these investigated a limited number of clinical phenotypes. There may also be correlations unique to the Canadian population that would otherwise not be observed from a global database. We particularly examined the relationships between patient genotype and clinical diagnosis (DMD/BMD), as well as between patient genotype and clinical outcomes (e.g., wheelchair use and cardiomyopathy status). We also determined the applicability of recent U.S. Food and Drug Administration (FDA)-approved exon skipping DMD therapeutics to the CNDR DMD patient population, given the increasing entry of this class of therapies into the clinic. Finally, this work provides the most recent characterization of the *DMD* mutation landscape in Canada.

## 2. Materials and Methods

### 2.1. Study Population and Design

This study was approved by the University of Alberta Health Research Ethics Board—Health Panel (reference Pro00092569). Participants in the CNDR provided informed consent and agreed to have their data shared for research purposes. For this study, the following information was used from CNDR patient records, which were provided directly from the clinic by neuromuscular specialists in the CNDR network: weight, height, clinical diagnosis, genetic data (test information, mutation type, mutation location), neuromuscular data (motor function, therapies received), cardiac history (presence of cardiomyopathy, left ventricle ejection fraction (LVEF), cardiac medications received), respiratory data (use of non-invasive/invasive ventilation, forced vital capacity (FVC)), and gastrointestinal data (feeding tube use, major nutritional route). Clinical diagnosis (DMD/BMD) was at the discretion of the neuromuscular specialist attending to the patient on the basis of clinical and genetic characteristics. All genetic data were derived from accredited testing laboratories across Canada as part of standard clinical practice. If a patient had information in the registry from more than one visit, data from the most recent visit was considered for analysis. All patient data were de-identified before provision to the study team.

**Figure 1:**
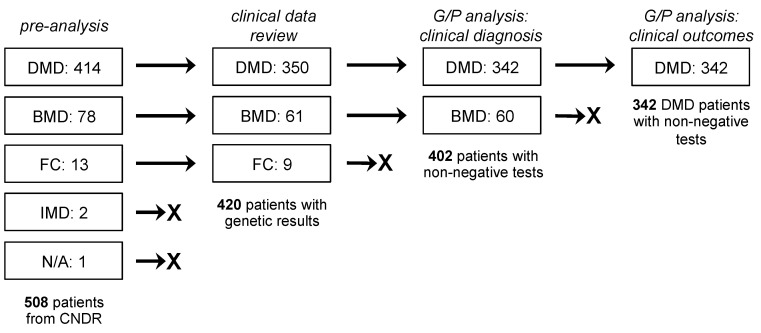
Study population and design. Patient data from the Canadian Neuromuscular Disease Registry between January 2012 and July 2019 were used for this study. The number and groups of patients evaluated for the various analyses performed are shown. DMD, Duchenne muscular dystrophy; BMD, Becker muscular dystrophy; FC, female carrier; IMD, intermediate muscular dystrophy; G/P, genotype–phenotype.

The initial study population consisted of 508 dystrophinopathy patients in the CNDR from 1 January 2012 to 3 July 2019. This included 414 DMD patients, 78 BMD patients, 13 female *DMD* mutation carriers, 2 intermediate muscular dystrophy (IMD) patients, and 1 with an unknown diagnosis (Figure 1). We filtered out patients who did not have genetic testing data or a definite DMD/BMD diagnosis, leaving us with 420 patients (350 DMD patients, 61 BMD patients, 9 female carriers). Data from these patients were used for comparisons of clinical outcomes across groups. For correlational analysis between genotype and clinical diagnosis as phenotype, we focused only on the 342 DMD and 60 BMD patients with non-negative genetic test results. On the other hand, for the analysis between genotype and clinical outcomes (wheelchair use, presence of cardiomyopathy, LVEF, FVC), we restricted our analysis to include only the 342 DMD patients.

### 2.2. Statistical Analysis

All statistical analyses and plotting were performed using GraphPad Prism version 8.4.3 (GraphPad Software, San Diego, CA, USA). A two-sided Fisher’s exact test was done to determine statistically significant differences between groups of categorical variables, while a two-tailed, unpaired Student’s *t*-test was done for continuous variables. A multiple logistic or linear (least squares) regression analysis was used to construct inferential models studying the relationships between genotypes and clinical outcomes, with the latter serving as dependent variables. Patients with missing information were excluded from the multiple regression analyses by the software. A *p*-value of less than 0.05 was considered statistically significant.

## 3. Results

### 3.1. Clinical Characteristics

Table 1 summarizes the clinical characteristics of the three subgroups in our study population: DMD, BMD, and female carriers. The female carriers all appear to be healthy, at least based on the parameters reviewed. However, the low number of carriers in our cohort (*N* = 9) makes it difficult to accurately compare with other subgroups. Thus, we decided to perform a comparative analysis of clinical characteristics only between DMD and BMD patients.

The DMD patients in our population were significantly younger by 7 years (*p* < 0.0001; mean ages of 10.5 versus 17.9 years old, respectively) and had lower body mass indices (BMIs) by 3 points (*p* < 0.005; mean BMIs of 18.1 versus 21.3, respectively) than the BMD patients. As expected, DMD patients used the wheelchair significantly more than BMD patients (*p* < 0.005), required more support for walking (*p* < 0.05) or sitting (*p* < 0.05), and were mostly on deflazacort therapy (*p* < 0.05). In terms of cardiac outcomes, no significant differences in cardiomyopathy status between DMD and BMD patients were observed in our population. However, the age of cardiomyopathy onset was significantly earlier for DMD at an average of 13.0 years than BMD at an average of 23.0 years (*p* < 0.05). Despite LVEF values being significantly lower in BMD than DMD patients (*p* < 0.05), both subgroups were well within the healthy LVEF range at >50%. These LVEF results likely reflect how patients from both groups also received standard cardiac medications in the form of angiotensin-converting enzyme inhibitors, angiotensin II-receptor blockers, and β-blockers, among others. FVC values were significantly reduced in DMD than in BMD patients (*p* < 0.005; 76.0% versus 88.0% on average, respectively). Perhaps due to scarcity in the available data, no significant differences in other respiratory or gastrointestinal parameters were found between the two patient subgroups.

### 3.2. Genetic Characteristics

**Figure 2:**
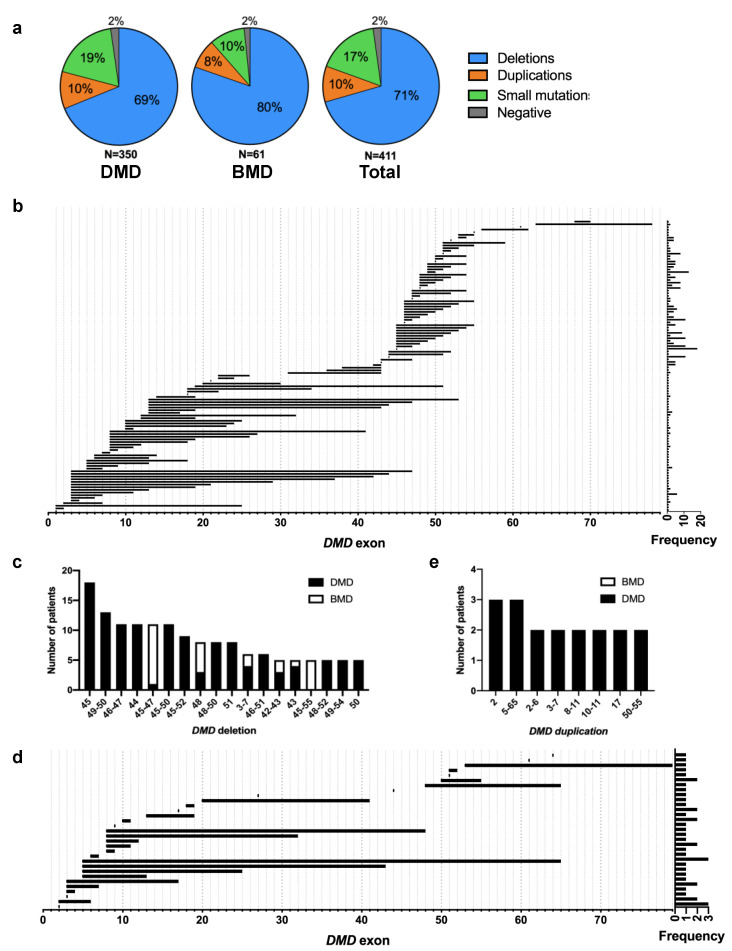
Overview of genetic characteristics in the study population. (**a**) *DMD* mutations in patients grouped according to type (deletions, duplications, small mutations); (**b**) Map of large *DMD* deletions (>1 exon) in Duchenne and Becker muscular dystrophy patients (DMD, BMD), with their frequencies (# patients) on the right (*N* = 290); (**c**) Top 18 most common large *DMD* deletions in DMD and BMD patients; (**d**) Corresponding map of large *DMD* duplications (>1 exon) in DMD and BMD patients (*N* = 41); (**e**) Top 8 most common large *DMD* duplications in DMD and BMD patients.

Genetic testing data was available for 350 of 414 DMD patients (85%) and 61 of 78 BMD patients (78%) (Figure 1). The majority of mutations were deletions of at least one exon in the *DMD* gene in 69% (241/350) of DMD patients and 80% (49/61) of BMD patients, or 71% (290/411) of patients in total (Figure 2a). This was followed by small mutations, i.e., point mutations and insertions/deletions within exons or splice sites, in 17% (71/411) of patients, and duplications of at least one exon in 10% (41/411) of patients. Negative results were found for 2% of patients, i.e., these patients were clinically diagnosed as having DMD/BMD, but genetic testing failed to identify a variant. However, as these patients were also not tested via gene sequence analysis, it remains possible that they could have deep intronic mutations in the *DMD* gene that were missed.

Mapping out all large deletions (>1 exon) revealed two mutation hotspots, one from exons 3 to 20 and another from exons 45 to 55 (Figure 2b). More than half of all patients with deletions at ~65% had mutations in the distal hotspot, whereas only ~21% were in the proximal hotspot. Moreover, most deletions in the proximal hotspot were represented by only one patient. The most common deletion was a deletion of exon 45, which was in 18 out of 290 patients (6%) with large deletion mutations (Figure 2c). Out of the 18 most common large deletion mutations, 17 were in the distal exons 45–55 mutation hotspot. Conversely, mapping out all large duplications (>1 exon) in our DMD and BMD patients revealed one hotspot from exons 3–10 (Figure 2d). However, note that most exon duplication patterns were represented by only one patient. The most common duplications were an exon 2 duplication and an extensive exons 5–65 duplication, which were each found in 3 out of 41 patients (7%) with large *DMD* duplication mutations (Figure 2e).

**Figure 3:**
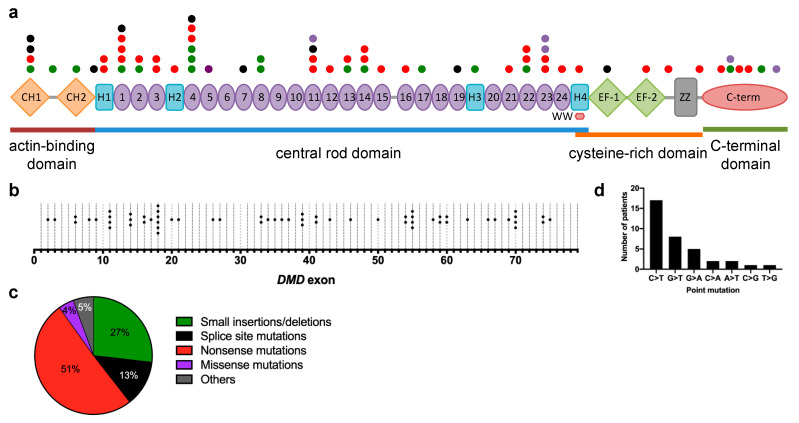
Overview of small mutations in the study population. (**a**) The positions of small mutations identified in Duchenne and Becker muscular dystrophy (DMD, BMD) patients are shown according to the domain/region of the dystrophin protein they affect, with each dot representing a unique mutation. The color of the dots correspond to the legend in (**c**); (**b**) The positions of small mutations, shown according to the *DMD* exon they are located in; (**c**) Distribution of *DMD* small mutations according to type (small insertions/deletions, splice site mutations, nonsense mutations, missense mutations, others); (**d**) Frequency of point mutation types in DMD and BMD patients. (*N* = 71).

Small mutations were spread out across the entire gene, ultimately affecting all four major dystrophin protein regions: the N-terminal actin-binding domain (exons 2–8), the central rod domain (exons 8–61), the cysteine-rich domain (exons 63–69), and the C-terminal domain (exons 70–79) (Figure 3a,b). Exons were assigned to protein domains following information from the Leiden Muscular Dystrophy dystrophin page (https://www.dmd.nl/). Exon 18 harbored the greatest number of small mutations in our combined DMD and BMD population (Figure 3b). More than half (51%) of all identified small mutations were nonsense point mutations, followed by 27% being small insertions/deletions, 13% being splice site mutations, and 4% being missense mutations (Figure 3c). Interestingly, two DMD patients each carried two different small mutations—one with c.8729A>T and c.8734A>G (both missense mutations; reported in the LOVD to frequently co-segregate with each other and are classified as benign), and one with c.10127T > C (a missense mutation) and c.10133dup (a frameshifting insertion mutation). There was also one DMD patient who had both a duplication of exon 61 and a nonsense c.9100C > T point mutation; for purposes of this study, this patient was grouped with other duplication mutation carriers. A survey of nonsense point mutations in our population showed that 47% (17/36) involved a C-to-T transition (Figure 3d).

### 3.3. Relationships between Genotype and DMD/BMD Diagnosis as Phenotype

**Figure 4:**
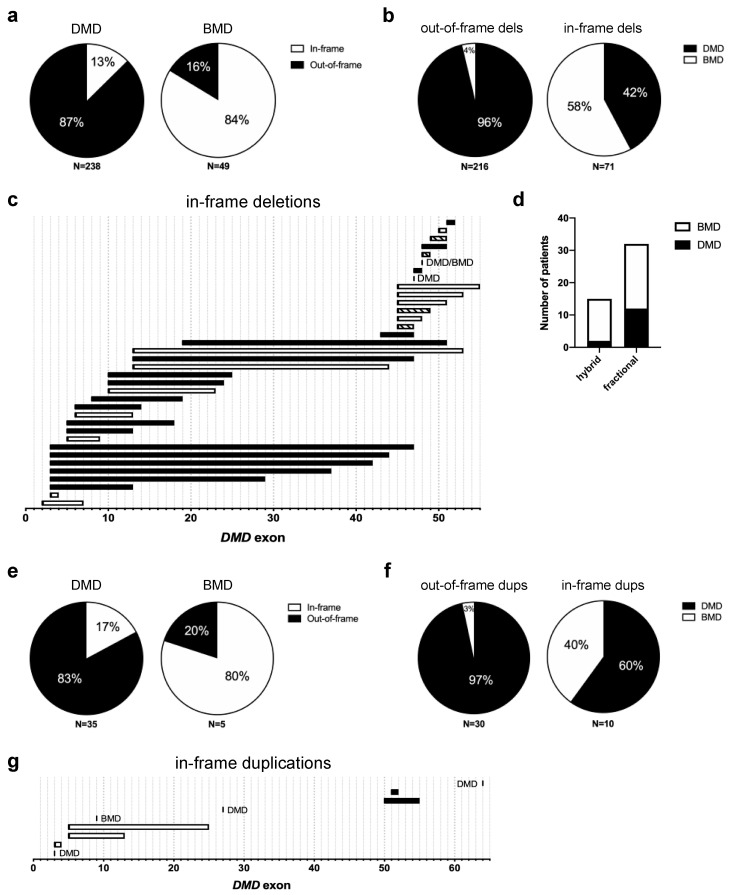
Analysis of large deletions and duplications, and their effect on the *DMD* reading frame. (**a**) Distribution of in-frame and out-of-frame deletions in Duchenne and Becker muscular dystrophy (DMD, BMD) patients; (**b**) Distribution of phenotypes associated with in-frame and out-of-frame deletions; (**c**) Map of in-frame *DMD* deletions in DMD and BMD patients, black: DMD, white: BMD, striped: both; (**d**) Frequencies of DMD and BMD patients with hybrid or fractional repeat-forming in-frame deletions; (**e**–**g**) Corresponding plots of (**a**–**c**) for duplications in DMD and BMD patients.

The reading frame rule predicts at least 90% of the time [11,22] if a given *DMD* mutation will lead to a DMD or BMD phenotype. Most out-of-frame mutations give rise to DMD, while most in-frame mutations give rise to BMD [8]. To determine how well this rule holds in our population, we examined the frequency of out-of-frame and in-frame deletions in our DMD and BMD patients from the CNDR (Figure S1a–c). Of the 238 DMD patients in our cohort with deletion mutations not involving either exon 1 or 79, 87% (208/238) had out-of-frame mutations and 13% (30/238) had in-frame mutations (Figure 4a). On the other hand, of the 49 BMD patients with corresponding deletions, 16% (8/49) had out-of-frame mutations and 84% (41/49) had in-frame mutations. 

Considering the deletions themselves, 96% (208/216) of observed out-of-frame deletions led to DMD, with only 4% (8/216) leading to BMD (Figure 4b). The in-frame deletions displayed a less skewed behavior—with 42% (30/71) giving rise to DMD and 58% (41/71) to BMD. Since the in-frame deletions did not predominantly favor one phenotype over the other to the same extent as out-of-frame deletions, we decided to map them out across the *DMD* exons. This will allow us to see if the location of the in-frame deletion is a key determinant of whether a patient develops DMD or BMD. The majority of in-frame deletions leading to DMD were found to start within the N-terminal exons 3–20 hotspot (Figure 4 and Figure S1b). In particular, of the 19 in-frame deletions solely associated with DMD, 14 or 74% of them started in this region. DMD-associated N-terminal in-frame deletions also tended to partially or completely remove more functional domains on the resulting dystrophin protein than their BMD-associated counterparts (Table S1). On the other hand, 67% (10/15) of in-frame deletions located at the distal half of the gene past exon 43 led to a BMD phenotype or to a mix of either a DMD or BMD phenotype (Figure 4c). 

As these distal in-frame deletions all occur within the central rod domain of the dystrophin protein, one could model in silico how well these preserve the filamentous, helical structure of the region. Depending on where the exon breakpoints are, an in-frame deletion can give rise to either a hybrid or a fractional repeat unit in the rod domain. Hybrid repeats maintain the filamentous structure of the rod domain, whereas fractional repeats disrupt it [23,24,25]. Using the eDystrophin database (http://edystrophin.genouest.org/) [25], we obtained modeling predictions for the repeat structures formed by the various distal in-frame deletions (Table 2). Although hybrid repeat-forming deletions were found in more BMD than DMD patients, no significant association was found between clinical phenotype (DMD/BMD) and the predicted repeat structure formed by an in-frame deletion in the exons 45–55 hotspot region (Figure 4d). Interestingly, despite giving rise to a predicted fractional repeat unit, the in-frame deletion of exons 45–47 led to BMD 91% of the time (10/11 patients) rather than DMD (Table 2).

We next examined the frequency of out-of-frame and in-frame duplications in our DMD and BMD patient population (Figure S2a,b). Of the 35 DMD patients in our cohort with duplication mutations, 83% (29/35) had out-of-frame mutations and 6% (6/35) had in-frame mutations (Figure 4e). Meanwhile, we only had five BMD patients with duplication mutations, one of which had an out-of-frame mutation, with the remaining four having in-frame mutations. In terms of the duplications themselves, out-of-frame duplications led to DMD 97% (29/30) of the time and to BMD 3% (1/30) of the time; in-frame duplications led to DMD in 60% (6/10) of cases and to BMD in 40% (4/10) of cases (Figure 4f). Similarly, as we did with the deletions, we mapped out all in-frame duplication patterns across the *DMD* exons (Figure 4g). Only nine unique in-frame duplications were found in our population, with those at the proximal end of the gene mostly associated with BMD and those at the distal end all associated with DMD.

Notably, less than 10% of small mutations (6/71) were associated with BMD in our study population. Due to the low representation of this mutation type among BMD patients, an analysis of genotype–phenotype correlations may be premature and therefore was not performed.

### 3.4. Relationships between Genotype and Clinical Outcome as Phenotype

We then proceeded to perform a series of multiple regression analyses to determine any relationships between patient genotypes and clinical outcomes, focusing on data from DMD patients (Figure 1). For genotype, we considered the location of the mutation according to which dystrophin protein domain/s or dystrophin isoform/s they affect. Exons were once again assigned to protein domains following information from the Leiden Muscular Dystrophy dystrophin page (https://www.dmd.nl/). For clinical outcomes, we looked at wheelchair use (combined permanent and intermittent use), cardiomyopathy status (presence or absence), LVEF, and FVC. In constructing these models, we also took into account the effect of other parameters such as age, BMI, steroid use (past or present), and use of cardiac medications, as appropriate. The results of these analyses are summarized in Table S2 and Table S3.

Multiple logistic regression analysis revealed that there is a 6.136 times increase in odds (95% confidence interval (CI): 1.44, 33.99; *p* < 0.05) that a DMD patient will require wheelchair use when they have mutations affecting the dystrophin rod domain (Table S2). Mutations affecting the C-terminal domain yielded an odds ratio of 0.0281 (95% CI: 0.001, 0.30; *p* < 0.005), indicating that their presence was associated with decreased wheelchair use in our DMD patient population. A similar relationship was found for mutations affecting the Dp116 and Dp71 isoforms (both *p* < 0.005). Across all models with wheelchair use as the selected outcome, age had an odds ratio greater than 1.75 (*p* < 0.0005), and BMI as well as steroid use were not significant predictors. All area under the receiving operator curve (AUC) values were at least 0.93. When cardiomyopathy status was used as an outcome, only mutations affecting the Dp140 isoform showed a significant relationship, with an odds ratio of 0.3662 (95% CI: 0.14, 0.92; *p* < 0.05) (Table S2). Age gave an odds ratio of at least 1.31 (*p* < 0.0005), with BMI and steroid use not being significant predictors of cardiomyopathy status; AUC values were at least 0.83. Unfortunately, models could not be generated for the other genotype categories, as these groups did not have any patients with cardiomyopathy.

Multiple linear regression analysis with LVEF as the outcome yielded no genotypes as significant predictors (Table S3). Age, steroid use, and use of cardiac medications all yielded significant estimates (β) in the produced regression models (individual R^2^ > 0.3). Age and use of cardiac medications gave negative estimates (*p* < 0.0005 and *p* < 0.005, respectively), while steroid use gave positive estimates (*p* < 0.05). On the other hand, when FVC was used as an outcome, mutations in the C-terminal domain gave a significant β in the model at −19.24 (95% CI: −36.56, −1.91; *p* < 0.05). No other genotype categories yielded significant β values. Age and steroid use had significant estimates in all produced models for FVC (individual R^2^ values >0.4), with age having negative β values (*p* < 0.0005) and steroid use having positive β values (*p* < 0.005).

### 3.5. Applicability of Exon Skipping Therapy to DMD Patients in Canada

A particularly promising approach to treat DMD is exon skipping using small single-stranded nucleic acid analogues called antisense oligonucleotides (AOs). In this strategy, AOs are designed to bind specific splicing enhancer sequences in out-of-frame *DMD* exons by base pairing. This results in the exclusion of targeted exons from the final mRNA transcript, restoring the reading frame and thereby allowing for the synthesis of shorter, partially functional dystrophin proteins [26,27]. With the increasing number of exon skipping therapies entering the clinic and receiving FDA approval, we sought to determine their applicability to DMD patients in Canada. We evaluated the applicability of the top 10 single exon skipping strategies that can treat the most number of patients registered in the global TREAT-NMD DMD database [11], and we also evaluated two multiple exon skipping strategies that target exons within the *DMD* mutation hotspots [18]. Exon 51 skipping treated the most number of DMD patients with deletions at 17%, as well as the most number of DMD patients overall (with deletions, duplications, and small mutations) at 12.3% in our cohort, which was similar to the trend observed worldwide in a previous TREAT-NMD study [11] (Table 3). This was followed by exon 45 skipping at 15.8% of DMD patients with deletions or 11.1% of all DMD patients and then by exon 44 skipping at 12.9% of DMD patients with deletions or 9.4% of all DMD patients. Exon 53 skipping is only the fourth most applicable single exon skipping therapy in our cohort, as opposed to being ranked second among TREAT-NMD DMD patients [11]. For the multiple exon skipping strategies, exons 45–55 skipping was applicable to 66.8% of DMD patients with deletions or 50.9% of all DMD patients in Canada (Table 3). Exons 3–9 skipping was less applicable, at 7.9% of all DMD patients with deletions or 9.1% of all DMD patients.

## 4. Discussion

We characterized *DMD* mutation data from DMD/BMD patients registered in the CNDR between 2012 and 2019, with a subsequent analysis of genotype–phenotype correlations. This study partly builds on previous work done by the Canadian Pediatric Neuromuscular Group (CPNG) in 2011, who studied the spectrum of *DMD* mutations in 773 patients across Canada from 2000 to 2009 [16]. We observed a similar abundance of mutation types across patients as the CPNG, with deletions forming the largest group (71% here compared to 64% from the CPNG study), followed by small mutations and duplications (Figure 2a). We found similar *DMD* mutation hotspots, with the exception that the CPNG observed a more extensive duplication hotspot from exons 2–20. In terms of overall genetic characteristics, our findings were largely consistent with those from global database studies (TREAT-NMD, LOVD) [11,18], indicating underlying commonalities in *DMD* gene mutability between patients in Canada and the rest of the world.

Perhaps the most well-known genotype–phenotype correlation in the field concerns the reading frame rule [8]. As in other studies (e.g., [11,16,18,19]) we found exceptions to this rule, with only 87% of DMD patients in our population having out-of-frame deletions and 84% of BMD patients having in-frame deletions (Figure 4a), for a total exception rate of 13%, which was higher than what was observed in the TREAT-NMD and LOVD databases [11,18]. Examining the 36 in-frame deletion patterns in our cohort revealed that deletion location and size matter, particularly if it affects dystrophin protein-binding domains mostly concentrated at the N-terminal end of the protein (Figure 4c, Table S1). In-frame deletions within the rod domain-coding region past exon 45, which do not code for any known protein-binding domains, were mostly associated with BMD. However, the number of impacted binding domains does not completely predict the disease phenotype of in-frame deletions. Consider our in-frame deletions that start on exon 13: exons 13–44 and 13–53 deletions lead to BMD, while the sandwiched exons 13–47 deletion leads to DMD. All three affect the same dystrophin protein-binding domains (Table S1) and yet have varying clinical consequences.

It is possible that regions other than the currently known protein-binding domains may be more critical for dystrophin function. For instance, a previous study looked at 97 patients from the Universal Mutation Database (UMD)-DMD registry with in-frame deletions before exon 35 and suggested that certain protein-binding domains may be dispensable to dystrophin function [28]. Characterizing these other potential critical regions in the *DMD* gene would be essential to understanding patients with mutations not governed by the reading frame rule. These regions can be identified through a combination of extensive patient database study and in vitro validation with patient-derived cells or induced pluripotent stem cell-derived models [29] of patient mutations. The identification of such regions will also benefit the development of gene replacement or correction therapies for DMD [24] to ensure that the dystrophin protein variants used or produced by these approaches are as functionally close as possible to the full-length version.

One concern for in-frame deletions affecting the central rod domain is also whether or not they can preserve its repeating, filamentous structure. Intuitively, in-frame mutations that can maintain this structure would be more likely to lead to BMD. While we observed this to be somewhat true for hybrid repeat-forming deletions, the same surprisingly cannot be said for fractional repeat-forming deletions (Figure 4d). In fact, one study of LOVD patients with in-frame mutations between exons 42 and 57 even found that fractional repeat-forming deletions were more commonly associated with BMD (72% of the time) than DMD [24]. The same study showed that the position of in-frame mutations relative to hinge 3 (exons 50–51) better determines phenotype than the predicted repeat structure formed by the deletions, which is a finding corroborated by another report [30]. This suggests that other parameters should be considered when evaluating the consequences of in-frame mutations on dystrophin structure, such as effects on overall protein flexibility or intra-protein interactions between residues. However, it is important to point out that knowing this information would still not be sufficient to explain certain cases, such as why the same in-frame deletion leads to a mix of DMD and BMD patients (e.g., deletions of exons 45–47, 45–49, 48, 48–49, and 49–51; Figure 4c and Figure S1b). In these cases, genetic modifiers [31,32] or spontaneous exon skipping events (as discussed in the next paragraph) may play a role in determining patient phenotypes.

We also saw a few out-of-frame deletion patients in our cohort to be exceptions to the reading frame rule, particularly those with deletions in exons 3–6, 3–7, 3–21, 7–8, 42–43, and 43 (Figure S1b). Two mechanisms have been proposed to explain such exceptions. The first is the use of alternative translational start sites further downstream in the *DMD* transcript [33,34,35]. For instance, a series of immunofluorescence experiments performed on skeletal muscle biopsies from exons 3 to 7 deletion patients suggested that there was a potential alternative initiation codon in exon 8 [34]. Dystrophin was not detectable when antibodies recognizing the 5′ end of exon 8 in the protein were used; however, dystrophin was detected using antibodies recognizing the 3′ end of exon 8. This may explain why a deletion of exons 3–7 is typically associated with BMD or with milder DMD phenotypes [18,35,36]. The second mechanism is the occurrence of spontaneous exon skipping events that convert out-of-frame into in-frame mutations. A well-documented example is the spontaneous skipping of exon 44 that occurs when the exons flanking it are deleted [37,38]. In fact, exon 44-skippable deletions are usually associated with a higher number of dystrophin-revertant fibers and milder DMD phenotypes such as prolonged ambulation [36,39,40,41,42]. In addition, of the six out-of-frame deletions that we have listed as exceptions, five of them can be converted into in-frame deletions with the skipping of just one exon adjacent to the deletion. This spontaneous exon skipping may be tied to how the junction sequences formed by a deletion influences splicing, i.e., if it creates or destroys exon splicing silencer/enhancer sequences [37]. Further study into this phenomenon may also provide hints regarding the formation of dystrophin-revertant fibers.

As for correlations between genotypes and clinical outcomes, it is important to emphasize that the regression analysis performed here produces an inferential model, i.e., a model that best describes the study population at its current state. There were a number of limitations with the study population as it is now that may have affected the analysis, mostly concerning low sample sizes for each mutation pattern observed and incomplete availability of clinical outcome data for all patients. The majority of DMD patients analyzed were within the younger range as well (Table 1), and so there may be some bias in the observed phenotypes. For practical reasons, we also limited our analysis to genotypes classified according to the protein domain or the dystrophin isoform affected by the respective patient mutations. We acknowledge that use of other stratification procedures may lead to differing conclusions. 

With these in mind, we saw an increased likelihood of wheelchair use associated with mutations affecting the rod domain and, conversely, a decreased likelihood with mutations affecting the C-terminal domain and Dp116/71 isoforms in our DMD patient population (Table S2). It is interesting that a positive association with rod domain mutations was observed. Previous reports have shown that certain rod domain-coding mutations are associated with prolonged ambulation in DMD patients, e.g., exon 44-skippable deletions [36,40,41,42]. Once a sufficient number of patients are available, it may be worthwhile to further stratify rod domain mutations to pinpoint the importance of specific sub-regions. The finding regarding the C-terminal domain is striking, since one would expect it to be critical in localizing dystrophin to the muscle membrane [7]; note that C-terminal domain mutations were also significantly, positively correlated with FVC in our DMD patient cohort (Table S3). Interestingly, there has been a previous case of an 8-year-old boy reported to be asymptomatic despite having a nonsense mutation truncating the C-terminal domain [43]. Microdystrophins lacking most or all of the C-terminal domain have also been promising in *mdx* mice with improvements in skeletal and cardiac muscle phenotypes [44,45,46]. Our results complement such findings, inviting closer investigation into the importance of the C-terminal domain for dystrophin function in muscle. However, it is also important to note that our result is based on a small number of patients with C-terminal domain mutations (*n* = 10), and so further validation by conducting a regression analysis with a larger sample size is recommended.

The association of Dp116 and Dp71 with motor function was likewise unexpected, as these isoforms are not normally expressed in differentiated skeletal muscle. Dp116 is exclusively expressed in Schwann cells [47], and Dp71 displays mostly ubiquitous expression but is difficult to detect in differentiated skeletal muscle [48,49]. While some reports are now claiming otherwise [50,51], i.e., that these isoforms are in fact expressed in muscle (one study is described in the next section for Dp116), their functional significance in muscle remains unknown. As for other factors included in the model for wheelchair use, it was surprising that steroid use did not have a significant impact, contrary to a previous TREAT-NMD DMD registry report [17]. However, this observation may be restricted to the particular demographic of the population under study.

Mutations affecting Dp140 was the only genotype group determined to be a significant predictor of cardiomyopathy (Table S2); no significant genotypes were found as predictors for LVEF (Table S3). Dp140 is a non-muscle dystrophin isoform typically expressed in the central nervous system and the kidneys [52]; its expression in the heart (or skeletal muscles) has not yet been demonstrated. Based on our analysis, Dp140 mutations are apparently associated with the lack of cardiomyopathy. One group previously studied the relationship between cardiac dysfunction (LVEF <53%)-free survival and dystrophin isoform mutations, but they did not find any significant association with respect to Dp140 [51]. Instead, the authors observed that Dp116 mutations were significantly linked to better rates of cardiac dysfunction-free survival, which we did not see in our analysis. Note that Dp116 was thought to be a non-muscle dystrophin isoform; however, this study demonstrated that Dp116 mRNA expression was detectable in both human cardiac and skeletal muscle samples. Therefore, it remains possible that Dp140 may have a role in the heart, but this will have to be supported first by in vivo validation of cardiac Dp140 expression similar to what was done for Dp116 in the study above, and then by further confirmation of our result in other patient registries. Considering other factors in our model, steroid use was not a significant predictor for cardiomyopathy, but it was significantly, positively correlated with LVEF. This may be explained in part by the fact that our DMD patient cohort is relatively young and not well-suited for observing cardiac symptoms that manifest relatively late in the disease. Cardiac medications were significantly, negatively correlated with LVEF, but they may reflect the bias that patients with reduced LVEF are typically the ones receiving such treatments—the factor was included more as a control for other predictors.

There are other reports of genotype–phenotype correlations with respect to cardiac outcomes in the literature, with proximal/N-terminal mutations generally associated with worse cardiac symptoms than distal/C-terminal mutations [21,53,54,55]. Still, some studies demonstrated a lack of correlation altogether [21,56,57]. This issue of non-agreement across genotype–phenotype correlation studies is not only true for cardiac outcomes but also for skeletal muscle phenotypes. This clearly indicates the need for further work in this area, starting perhaps by standardizing data collection procedures to maximize comparability across patient registries as well as the amount of information obtained from each patient.

Within the last five years, we have seen the approval of three exon skipping AOs for DMD therapy by the FDA: eteplirsen (brand name Exondys 51, Sarepta) for skipping exon 51 in 2016 [26], and golodirsen (Vyondys 53, Sarepta) in 2019 [58] as well as viltolarsen (Viltepso, NS Pharma) in 2020 [59] for skipping exon 53; another AO, the exon 45-skipping casimersen (SRP-4045, Sarepta) is currently under FDA review. These FDA-approved therapies can treat a combined 26.5% of DMD patients with deletions or 19.3% of all DMD patients in Canada (Table 3), which is incredibly encouraging. Notably, the applicability of single exon skipping strategies was different for patients in Canada compared to global estimates from the TREAT-NMD DMD database [11], suggesting potential implications for future clinical trials. These findings highlight one of the major limitations associated with personalized therapies such as exon skipping, i.e., low patient applicability. One way to overcome this would be to develop multi-exon skipping strategies such as exons 45–55 skipping, which could treat more than half of all DMD patients (Table 3). Our data and those from other patient registries [18,60] also show that exons 45–55 deletions are commonly associated with mild BMD or asymptomatic phenotypes (Figure 4c and Figure S1b), confirming the viability of the approach as a treatment for DMD.

This last point raises a concern for other exon skipping strategies, i.e., if the in-frame-skipped dystrophin proteins they produce are indeed functional or associated with mild phenotypes. We have seen how some deletions lead to a DMD phenotype despite being in-frame, e.g., in our population, 42% of in-frame deletions were in DMD patients (Figure 4b). Encouragingly, the majority of patients with deletions equivalent to exon 51-skipped transcripts showed mild phenotypes [61], bearing well for eteplirsen. Therefore, consulting patient registries such as the CNDR when designing exon skipping strategies is recommended. Finally, despite the promise of exon skipping therapy, it cannot correct all mutations, and there remain concerns regarding its efficacy in patients. The continued development of other therapeutic approaches such as gene replacement with mini/microdystrophins or gene correction with genome editing strategies, as informed by genotype–phenotype correlation studies from patient registries, remains critically important.

## Figures and Tables

**Table 1:** Summary of clinical characteristics for patients with genetic data in our study population.

Characteristic	DMD ^1^	BMD ^1^	FC ^1^	*p*-Value ^2^
Number (*N*)	350 (83)	61 (15)	9 (2)	-
Age at visit (yr)	10.5 (6.8–14.6)	17.9 (14.1–24.9)	13.0 (11.9–15.0)	<0.0001
Body mass index	18.1 (16.2–22.8)	21.3 (17.6–26.6)	17.0 (15.4–25.0)	0.0093
**Neuromuscular parameters**				
Wheelchair use				0.0023
>Permanent	39 (11)	2 (3)	0 (0)	
>Intermittent	98 (28)	10 (16)	0 (0)	
>Never	156 (45)	43 (70)	8 (89)	
>Unknown	57 (16)	6 (10)	1 (11)	
Can walk without support	189 (62)	43 (78)	9 (100)	0.0175
Can sit without support	244 (81)	52 (95)	9 (100)	0.013
Uses steroids				0.0218 ^3^
>Deflazacort	231 (91)	6 (67)	1 (100)	
>Prednisone	19 (7)	3 (33)	0 (0)	
>Vamorolone	1 (0)	0 (0)	0 (0)	
>Testosterone	3 (1)	0 (0)	0 (0)	
**Cardiac parameters**				
Cardiomyopathy	37 (11)	10 (17)	0 (0)	0.1928
Age of CM onset (yr)	13.0 (11.0–14.3)	23.0 (16.0–33.0)	-	0.0059
Left ventricle ejection fraction (%)	63.0 (58.0–68.0)	60.0 (50.0–65.0)	68.5 (62.0–70.5)	0.0325
Uses cardiac medication				0.0197 ^4^
>ACEi/ARB	69 (70)	9 (43)	-	
>β-blocker	18 (18)	5 (24)	-	
>Digoxin	5 (5)	0 (0)	-	
>Statin	0 (0)	2 (10)	-	
>Antiplatelet	1 (1)	1 (5)	-	
>Anticoagulant	0 (0)	3 (14)	-	
>MRA	3 (3)	0 (0)	-	
**Respiratory parameters**				
Uses ventilation assistance				>0.9999
>Non-invasive	30 (9)	2 (3)	0 (0)	
>Invasive	1 (0)	0 (0)	0 (0)	
Forced vital capacity (%)	76.0 (55.0–93.0)	88.0 (80.0–100.0)	74.0 (59.0–85.3)	0.0018
Sleep apnea	10 (38)	0 (0)	0 (0)	0.3703
**Gastrointestinal parameters**				
Uses feeding tube	2 (1)	0 (0)	0 (0)	>0.9999
Major nutritional route				>0.9999
>Oral	132 (99)	16 (100)	2 (100)	
>Enteral	1 (1)	0 (0)	0 (0)	

^1^ count data: frequency (%), continuous data: median (interquartile range), ^2^ DMD versus BMD, ^3^ vamorolone and testosterone counted as one group, ^4^ digoxin up to MRA counted as one group. DMD, Duchenne muscular dystrophy; BMD, Becker muscular dystrophy; FC, female carrier.

**Table 2:** Repeat structure modeling of in-frame *DMD* deletions within the exons 45–55 hotspot.

In-Frame Deletion	Frequency DMD (%)	Frequency BMD (%)	Predicted Repeat Structure ^1^
45–47	1 (9.1)	10 (90.9)	Fractional
45–48	0 (0)	4 (100)	Hybrid
45–49	1 (50)	1 (50)	Fractional
45–51	0 (0)	2 (100)	Hybrid
45–53	0 (0)	1 (100)	Hybrid
45–55	0 (0)	5 (100)	Hybrid
47	2 (100)	0 (0)	Fractional
47–48	1 (100)	0 (0)	Hybrid
48	3 (37.5)	5 (62.5)	Fractional
48–49	1 (33.3)	2 (66.7)	Fractional
48–51	2 (100)	0 (0)	Fractional
49–51	1 (50)	1 (50)	Hybrid
50–51	0 (0)	2 (100)	Fractional
51–52	2 (100)	0 (0)	Fractional

^1^ Information obtained from the online eDystrophin database.

**Table 3:** Applicability of single and multiple exon skipping strategies to DMD patients in Canada.

Exon/s to Skip	% of DMD Patients with Deletions	% of all DMD Patients	Rank in TREAT-NMD ^1^	Rank in Our Population
51	17.0	12.3	1	1
53	9.5	7.0	2	4
45	15.8	11.1	3	2
44	12.9	9.4	4	3
43	5.0	4.1	5	7
46	7.9	5.8	6	5
50	5.0	3.8	7	8
52	3.7	2.6	8	10
55	5.0	4.7	9	6
8	2.9	3.5	10	9
45–55	66.8	50.9	n/a	n/a
3–9	7.9	9.1	n/a	n/a

^1^ Rank information obtained from Bladen et al. (2015) [11].

## Supplementary Materials

The following are available online at https://www.mdpi.com/2075-4426/10/4/241/s1, Figure S1: Summary of large *DMD* gene deletions, Figure S2: Summary of large *DMD* gene duplications, Table S1: In-frame deletions and their effects on dystrophin protein-binding domains, Table S2: Multiple logistic regression analysis for wheelchair use and cardiomyopathy status, Table S3: Multiple linear regression analysis for left ventricle ejection fraction (LVEF) and forced vital capacity (FVC).
