# Supplementary material for: Genotype–Phenotype Correlations in Duchenne and Becker Muscular Dystrophy Patients from the Canadian Neuromuscular Disease Registry
Source: J Pers Med. 2020 Nov 23;10(4):241. doi: 10.3390/jpm10040241 (PMC7712074; doi:10.3390/jpm10040241)
Supplement: Supplementary file 1 [file jpm-10-00241-s001.pdf]

Supplementary Material

Supplementary Figures

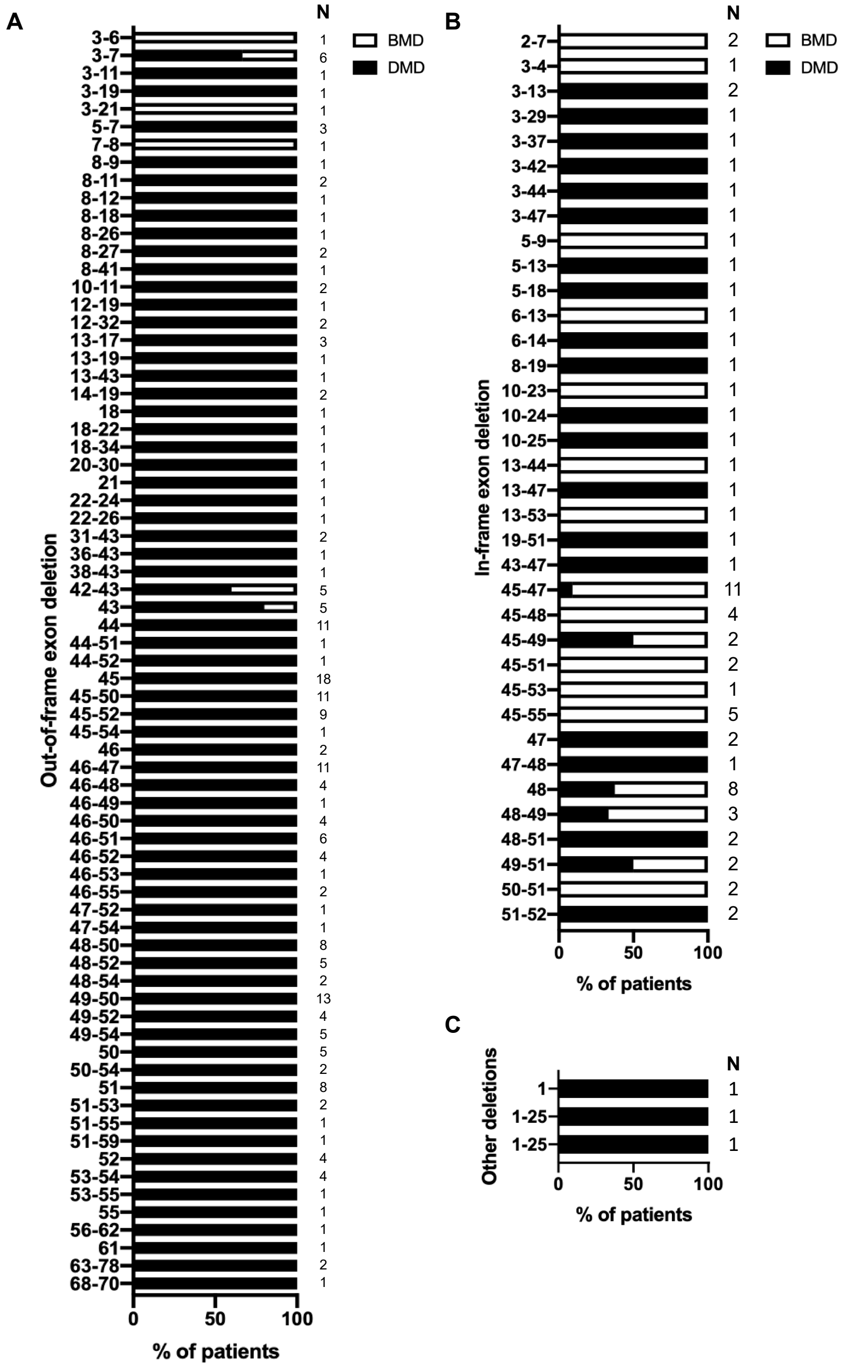

Figure S1. Summary of large *DMD* gene deletions. Shown are all large **(a)** out-of-frame, **(b)** in-frame, and **(c)** other deletion patterns with their respective distributions in Duchenne and Becker muscular dystrophy (DMD, BMD) patients in our study population.

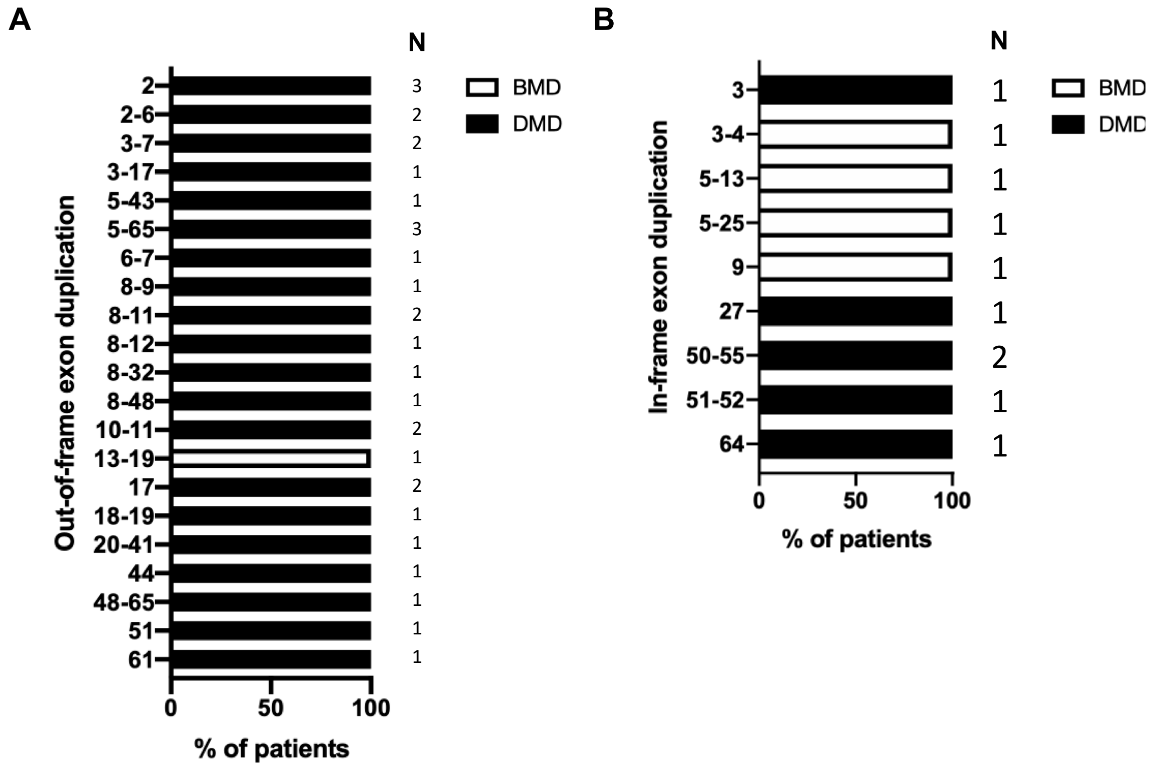

Figure S2. Summary of large *DMD* gene duplications. Shown are all large **(a)** out-of-frame and **(b)** in-frame duplication patterns with their respective distributions in Duchenne and Becker muscular dystrophy (DMD, BMD) patients in our study population.

Supplementary Tables

Table S1. In-frame deletions and their effects on dystrophin protein-binding domains[1].

In-frame deletion[2]	K19 (2-8)	ABD1 (2-8)	LBD1 (10-16)	LBD2 (17-50)	PAR-1b (26-30)	ABD2 (32-45)	Synemin (32-40)	nNOS (42-45)
2-7	partial	partial						
3-4	partial	partial						
3-13	partial	partial	partial					
3-29	partial	partial	lacking	partial	partial			
3-37	partial	partial	lacking	partial	lacking	partial	partial	
3-42	partial	partial	lacking	partial	lacking	partial	lacking	partial
3-44	partial	partial	lacking	partial	lacking	partial	lacking	partial
3-47	partial	partial	lacking	partial	lacking	lacking	lacking	lacking
5-9	partial	partial						
5-13	partial	partial	partial					
5-18	partial	partial	lacking	partial				
6-13	partial	partial	partial					
6-14	partial	partial	partial					
8-19	partial	partial	lacking	partial				
10-23			lacking	partial				
10-24			lacking	partial				
10-25			lacking	partial				
13-44			partial	partial	lacking	partial	lacking	partial
13-47			partial	partial	lacking	lacking	lacking	lacking
13-53			partial	lacking	lacking	lacking	lacking	lacking
19-51				partial	lacking	lacking	lacking	lacking
43-47				partial		partial		partial
45-47				partial		partial		partial
45-48				partial		partial		partial
45-49				partial		partial		partial
45-51				partial		partial		partial
45-53				partial		partial		partial
45-55				partial		partial		partial
47				partial				
47-48				partial				
48				partial				
48-49				partial				
48-51				partial				
49-51				partial				
50-51				partial				
51-52								

[1] Information on domains from the eDystrophin database (http://edystrophin.genouest.org/), empty cells indicate that the domain is not affected by the mutation; exons, in parentheses, assigned to domains using information from the Leiden Muscular Dystrophy page (https://www.dmd.nl/), [2] Cell color indicates which patients the in-frame deletion has been observed in: white, Becker muscular dystrophy; black, Duchenne muscular dystrophy; gray, both patients

Table S2. Multiple logistic regression analysis for wheelchair use and cardiomyopathy status.

Outcome	Mutation location[1] / isoform affected[2]	Odds ratio (95% CI)	Other odds ratios (95% CI)	AUC
Wheelchair use (permanent + intermittent)	A	ns	1.757*** (1.55,2.05) Age; ns BMI/Steroids	0.9330
	B	6.136* (1.44,33.99)	1.794*** (1.57,2.11) Age; ns BMI/Steroids	0.9408
	C	ns	1.774*** (1.56,2.08) Age; ns BMI/Steroids	0.9398
	D	0.0281** (0.001,0.30)	1.829*** (1.59,2.17) Age; ns BMI/Steroids	0.9382
	Dp260	ns	1.758*** (1.55,2.06) Age; ns BMI/Steroids	0.9317
	Dp140	ns	1.757*** (1.54,2.05) Age; ns BMI/Steroids	0.9322
	Dp116	0.0910** (0.02,0.42)	1.831*** (1.59,2.18) Age; ns BMI/Steroids	0.9398
	Dp71	0.0211** (0.001,0.18)	1.851*** (1.60,2.21) Age; ns BMI/Steroids	0.9431
	Dp40	ns	1.774*** (1.56,2.08) Age; ns BMI/Steroids	0.9398
Cardiomyopathy	A	ns	1.314*** (1.20,1.46) Age; ns BMI/Steroids	0.8364
	B	ns	1.328*** (1.21,1.48) Age; ns BMI/Steroids	0.8514
	C	not possible		
	D	not possible		
	Dp260	ns	1.310*** (1.20,1.45) Age; ns BMI/Steroids	0.8367
	Dp140	0.3662* (0.14,0.92)	1.316*** (1.20,1.46) Age; ns BMI/Steroids	0.8516
	Dp116	not possible		
	Dp71	not possible		
	Dp40	not possible		

[1] Letters indicate which protein domain is affected by the mutation: A, actin-binding domain (exons 2-8, N=40); B, rod domain (exons 8-61, N=304); C, cysteine-rich domain (exons 63-69, N=15); D, C-terminal domain (exons 70-79, N=10), [2] Dp260 (exons 30-79, N=250), Dp140 (exons 45-79, N=203), Dp116 (exons 56-79, N=28), Dp71 (exons 63-79, N=20), Dp40 (exons 63-69, N=14). *p<0.05, **p<0.005, ***p<0.0005; (N=342 DMD patients); ns, not significant; BMI, body mass index; CI, confidence interval; AUC, area under the receiver operating curve

Table S3. Multiple linear regression analysis for left ventricle ejection fraction (LVEF) and forced vital capacity (FVC)

[1] Letters indicate which protein domain is affected by the mutation: A, actin-binding domain (exons 2-8, N=40); B, rod domain (exons 8-61, N=304); C, cysteine-rich domain (exons 63-69, N=15); D, C-terminal domain (exons 70-79, N=10), [2] Dp260 (exons 30-79, N=250), Dp140 (exons 45-79, N=203), Dp116 (exons 56-

Outcome	Mutation location[1] / isoform affected[2]	β (95% CI)	Other Estimates (95% CI)	R^2
LVEF	A	ns	-1.000*** (-1.26,-0.74) Age; ns BMI; 4.226* (0.94,7.52) Steroids; -4.105** (-6.94,-1.27) Cardiac meds	0.3699
	B	ns	-1.011*** (-1.27,-0.75) Age; ns BMI; 4.023* (0.71,7.33) Steroids; -3.922** (-6.79,-1.06) Cardiac meds	0.3612
	C	ns	-1.006*** (-1.26,-0.75) Age; ns BMI; 4.211* (0.90,7.53) Steroids; -4.213** (-7.09,-1.33) Cardiac meds	0.3630
	D	ns	-1.014*** (1.27,-0.76) Age; ns BMI; 4.108* (0.78,7.43) Steroids; -4.023** (-6.88,-1.17) Cardiac meds	0.3601
	Dp260	ns	-1.021*** (-1.28,-0.76) Age; ns BMI; 4.250* (0.94-7.56) Steroids; -4.133** (-6.98,-1.28) Cardiac meds	0.3655
	Dp140	ns	-1.009*** (-1.27,-0.75) Age; ns BMI; 4.039* (0.74-7.34) Steroids; -4.109** (-6.96,-1.26) Cardiac meds	0.3634
	Dp116	ns	-1.010*** (-1.27,-0.75) Age; ns BMI; 4.258* (0.93,7.59) Steroids; -4.205** (-7.09,-1.32) Cardiac meds	0.3626
	Dp71	ns	-1.010*** (-1.27,-0.75) Age; ns BMI; 4.263* (0.94,7.58) Steroids; -4.179** (-7.04,-1.32) Cardiac meds	0.3638
	Dp40	ns	-1.008*** (-1.27,-0.75) Age; ns BMI; 4.207* (0.89,7.52) Steroids; -4.192** (-7.07,-1.32) Cardiac meds	0.3422
FVC	A	ns	-3.514*** (-4.12,-2.91) Age; ns BMI; 14.77** (4.05,25.49) Steroids	0.4253
	B	ns	-3.513*** (-4.12,-2.91) Age; ns BMI; 14.78** (4.07,25.49) Steroids	0.4265
	C	ns	-3.548*** (-4.15,-2.95) Age; ns BMI; 15.40** (4.77,26.04) Steroids	0.4365
	D	-19.24* (-36.56,-1.91)	-3.559*** (-4.16,-2.96) Age; ns BMI; 15.28** (4.68,25.88) Steroids	0.4391
	Dp260	ns	-3.508*** (-4.12,-2.90) Age; ns BMI; 14.50** (3.73,25.27) Steroids	0.4253
	Dp140	ns	-3.523*** (-4.12,-2.92) Age; ns BMI; 15.04** (4.40,25.69) Steroids	0.4335
	Dp116	ns	-3.521*** (-4.12,-2.92) Age; ns BMI; 15.35** (4.66,26.05) Steroids	0.4312
	Dp71	ns	-3.538*** (-4.14,-2.93) Age; ns BMI; 15.31** (4.64,25.98) Steroids	0.4326
	Dp40	ns	-3.552*** (-4.15,-2.95) Age; ns BMI; 15.31** (4.66,25.96) Steroids	0.4607

79, N=28), Dp71 (exons 63-79, N=20), Dp40 (exons 63-69, N=14). *p<0.05, **p<0.005, ***p<0.0005; (N=342 DMD patients); ns, not significant; BMI, body mass index; CI, confidence interval
